# The WISEWOMAN Program: Reflection and Forecast

**Published:** 2008-03-15

**Authors:** Julie C Will, Ryan K Loo

**Affiliations:** Centers for Disease Control and Prevention, National Center for Chronic Disease Prevention and Health Promotion; Centers for Disease Control and Prevention, National Center for Chronic Disease Prevention and Health Promotion, Atlanta, Georgia

## Abstract

The WISEWOMAN program targets low-income under- and uninsured women aged 40–64 years for screening and interventions aimed at reducing the risk of heart disease, stroke, and other chronic diseases. The program enters its third phase on June 30, 2008. Design issues and results from Phase I and Phase II have been published in a series of papers. We summarize remaining challenges, which were identified through systematic research and evaluation. Phase III will address these challenges through a number of new initiatives such as allowing interventions of different intensities, taking advantage of resources for promoting community health, and providing evidence-based interventions through the program's Center of Excellence. Finally, we provide a framework and vision so that organizational, community, and other partners can make the case for the importance of the program to their communities and for what is needed to make it work.

## Background

### Lessons learned

The Well-Integrated Screening and Evaluation for Women Across the Nation (WISEWOMAN) (1,2) program, funded by the Centers for Disease Control and Prevention (CDC), targets low-income under- and uninsured women aged 40–64 years. Aimed at reducing the risk of heart disease, stroke, and other chronic diseases, WISEWOMAN provides screening for high blood pressure, hypercholesterolemia, and abnormal glucose levels and interventions to help women eat more healthfully, increase physical activity, and quit smoking. The program refers women with abnormal screening values for treatment and at 1-year follow-up visits provides feedback to participants and their providers about changes in risk factor profiles (2). WISEWOMAN, which works in conjunction with the National Breast and Cervical Cancer Early Detection Program (NBCCEDP), has now reached more than 75,000 women.

Phase I of WISEWOMAN (1995–1998), which focused on research only, tested whether enhanced lifestyle interventions were more effective than lifestyle interventions using minimal or usual care. Design issues and results from this phase have been covered in a series of articles (3-6). During Phase II (1999–2007), state and tribal organizations could apply for either research funding or standard project funding, which allowed more flexibility in adapting previously tested, evidence-based interventions to local settings and made fewer research demands. Fifteen projects are now funded (Table 1), and results and overviews of 12 of these have been published (2,7-12). Lessons learned from Phase I (13) and Phase II are briefly summarized in Table 1. Implementation of Phase III is scheduled to begin June 30, 2008. We focus here on the use of lessons learned as the basis for planning the future of WISEWOMAN.

### Methods used to identify lessons learned

WISEWOMAN has identified lessons learned through randomized controlled trials (5,6), nonrandomized group-assigned trials (3), quasi-experimental studies such as pretest and posttest comparisons (8,9), cross-sectional studies employing benchmarks to measure performance (CDC, unpublished report, 2007), and case studies involving interviews with key stakeholders (11,13). Case studies were often mixed with analyses of benchmarks or outcome data to provide a richer description than could be obtained from quantitative data alone (11).

In Phase II, performance indicators for nonresearch projects included screening 2500 women annually and ensuring that 75% of screened participants began the lifestyle intervention, that 60% of women starting the intervention completed it, that 75% of women screened were rescreened within 10–14 months, and that 95% of women with systolic blood pressure >180 mm Hg or diastolic blood pressure >110 mm Hg were seen within 1 week of screening. Programs varied substantially in their ability to meet performance indicators. For example, the number of annual screenings performed by programs ranged from 449 to 5629, and initiation of lifestyle interventions ranged from 16% of participants in the lowest-performing program to 100% in the highest-performing program (CDC, unpublished report, 2007). Although the highest-performing programs demonstrated that most of the performance indicators are achievable, the WISEWOMAN program decided to revisit the feasibility of using these indicators and to identify obstacles to reaching them.

In an example of the mixed-methods approach, investigators conducted case studies of the sites that had the highest and the lowest public health impact within a select number of states and tribes (11,12). From this analysis, investigators identified practices that appeared to increase the chances of a successful project. These practices are described in the WISEWOMAN Best Practices Toolkit (www.cdc.gov/wisewoman).

WISEWOMAN participants who returned for 1-year evaluations showed significant reductions in systolic blood pressure (−0.9%. *P* < .05), total cholesterol (−1.8%, *P* < .05), smoking (−8.0%, *P* < .05), and 10-year risk of coronary heart disease (−4.9%, *P* < .05) (CDC, unpublished data, 2007). In the subgroup with abnormal screening values at baseline, these reductions were even more striking (8). In contrast, changes in weight and blood glucose values were both clinically and statistically insignificant, highlighting a need to strengthen the program in those areas (8). The importance of these risk factors is underscored by findings from the Diabetes Prevention Program Research Group (14), which showed weight loss and improved nutrition and physical activity to be more likely than medication alone to delay the onset of diabetes.

As a result of lessons learned in the previous phase, Phase II projects were strongly encouraged to use the socioecologic model (15), which includes the strengthening of linkages at the community and organizational levels. Success stories have been used as an additional method for identifying these types of lessons learned (16-18).

## Key Challenges

Phase III will address the following challenges that were identified in phases I and II:

- Maximize the number of women who receive program services (e.g., screening, lifestyle interventions, referral, follow-up).
- Target geographic areas in each state and tribe with the highest death rates and hospital discharge rates for heart disease and stroke.
- Deliver effective behavioral counseling based on sound theoretical approaches.
- Tailor lifestyle interventions according to degrees of risk for heart disease and stroke and readiness-to-change behaviors.
- Maximize the number of women being reassessed to determine whether their risk for heart disease and stroke has decreased and their health behaviors have improved.
- Collaborate with state, local, and community partners who can make policy, environmental, and system changes that support the adoption and maintenance of heart-healthy behaviors by underserved populations where they work, live, and play.

These challenges will be addressed through the requirements published in funding announcements, stronger program guidance, clarified program vision and goals, new performance indicators, and evidence-based technical assistance and training. As in the past, WISEWOMAN will use a variety of evaluation methods to determine the degree to which these key challenges have been met.

## Solutions for the Future

Most of the remainder of this article addresses two specific priorities: increasing the reach of the WISEWOMAN program and ensuring the effectiveness of its projects. Reach and effectiveness are the two criteria most directly related to determining WISEWOMAN's impact and thus merit extensive discussion. First, however, we focus on the importance of having a clear programmatic vision.

### Clarifying the vision

Recent evidence from implementation of the Chronic Care Model (19,20) suggests that multilevel approaches such as WISEWOMAN's work better than approaches that focus on one level alone. Legislative language, however, stipulates that 60% of dollars given to WISEWOMAN projects must be used for services to individual participants. Consequently, implementing the program at multiple levels is an ongoing challenge. Creating strong partnerships and links with organizations and communities to establish changes in the health system and in policy is key to solving this challenge.

The WISEWOMAN program employs the models used by Abrams et al (21) and Glasgow et al (22) to evaluate public health impact. In its early phases, WISEWOMAN tended to focus heavily on the effectiveness of a lifestyle intervention (e.g., 1-year reductions in blood pressure and cholesterol), but as the program expanded into nonresearch settings, projects began requesting a wider definition of success. Two appropriate new measures are the public health impact of a project and its value. Impact can be defined as either reach multiplied by effectiveness (21) or a variant that adds dimensions of adoption, implementation, and maintenance (22). Maximum impact is most likely when a large number of participants achieve small to moderate improvements in risk factors (i.e., effectiveness) than when fewer participants achieve greater improvements. A criterion of effectiveness alone is inappropriate for WISEWOMAN, because a program might be extremely effective but serve only a small percentage of eligible women (i.e., have limited reach). Value, the second measure of success, which we define as public health impact at a reasonable cost, is similar to cost-effectiveness, except that we measure the cost of achieving a certain impact (21) rather than effectiveness.

Another issue to be clarified involves approaches to implementing an intervention. Projects were required to select an evidence-based intervention, but ongoing guidance was not provided on tailoring interventions to new target populations or unique settings. Consequently, many projects did not faithfully implement the elements of their intervention that were credited with its effectiveness. Faithfully implementing these core elements is important, as demonstrated by the translation work of CDC's Division of HIV/AIDS Prevention, the Center on AIDS and Community Health, and the Academy for Educational Development (23) (www.effectiveinterventions.org).

**Figure 1.**
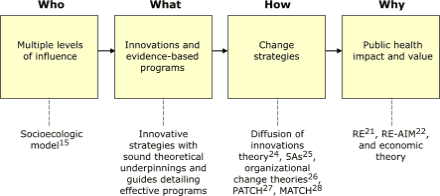
Organizing framework, WISEWOMAN, United States.

The WISEWOMAN program is complex, drawing on multiple theoretical models including the ecologic approaches defined in the socioecologic model; change theories aimed at altering behavior and organizational and community practices; and models that underscore the importance of creating public health impact through increased reach and effectiveness. Although WISEWOMAN stressed all of these models in the past, the program did not have an organizing framework showing how the various models related to each other. The new framework (Figure 1), which brings together these models, can be used by any public health program aimed at changing behavior and policy. As indicated in the figure, WISEWOMAN will use the socioecologic framework to identify partners working at individual, organizational, community, and state levels to ensure that all are working to maximize public health impact. The program is committed to evidence-based interventions and will advise projects on creating change at the behavioral, organizational, community and environmental, and policy levels. Finally, WISEWOMAN will expand its focus from the effectiveness of interventions alone to include public health impact and the cost of achieving such impact (i.e., value).

**Table:** 

From the beginning, the WISEWOMAN program has been delivered as a multicomponent intervention that comprises 1) screening of risk factors for cardiovascular disease and other chronic diseases, 2) lifestyle interventions, 3) assurance of access to needed treatment and medication, and 4) follow-up visits for monitoring and evaluation. These components are required by congressional legislation. The clinical guidelines that are the source of the evidence for each of these four components (Table 2) include the use of a multicomponent approach for maximum clinical effectiveness. Instead of focusing on determining which component works best, WISEWOMAN is committed to strengthening all four components.

### Maximizing the program's reach

One approach to expanding the reach of WISEWOMAN is to better match participants' needs with the program resources. Various interventions tailored to participants' degrees of risk or readiness to change might be the best way to proceed, or interventions might be designed to reduce costs per woman served. During Phase II, CDC asked that all participants be required to attend the same lifestyle intervention regardless of how strong their risk factors were or how ready they were to change. For many reasons, however, designing only one intervention for all women can limit a program's reach. Furthermore, women with normal screening values will most likely not need an intensive intervention designed to control risk factors, nor is it reasonable or cost-effective to ask a woman who is not ready to change to participate in a costly intervention. Referring women with highly abnormal screening values to low-intensity interventions that may not help control their risk factors is also likely to be ineffective.

Because women often describe time as the major barrier to participation in public health programs (29), asking them to invest time in a program unnecessarily is not reasonable. Abrams et al (21) have proposed a stepped-care approach in which participants begin with the least intensive intervention necessary, and only those whose risk is not reduced are offered more intensive interventions. Abrams et al also discuss an approach that matches programs to women by their readiness to change and the extent of their risk factors. Other behavioral scientists have suggested a variation of this approach, which is usually called triaging (30). Beginning in June 2008, the WISEWOMAN program will ask state and tribal programs to use these and other new models, including changing the mode of delivery to include approaches such as self-help; video-, computer-, or Web-based delivery; face-to-face assessments; and group counseling.

### Ensuring the effectiveness of strategies for change

Scientists have debated the degree to which every aspect of an intervention must be delivered exactly as it was in the randomized trial that demonstrated its effectiveness (31). An extreme argument is that any change to the original intervention, including translating it into another language and making it culturally appropriate, should require a new randomized trial, even if the core elements remain the same. Other scientists contend that if the core elements remain intact, interventions can be adapted to ensure fit to the local context and setting (23). In the future, WISEWOMAN projects will use the latter approach as part of an effort to maximize both effectiveness and the ability to tailor the intervention for local settings. To accomplish this, we will review evidence-based interventions and identify the core elements that must be faithfully implemented and other characteristics that increase the probability of success. We will then disseminate intervention packages that can be downloaded from a Web site. Practitioners will be able to choose from a menu of WISEWOMAN Interventions with Sound Evidence (WISE Interventions, www.wiseinterventions.org). Evaluation will focus on monitoring the implementation of core elements.

**Figure 2.**
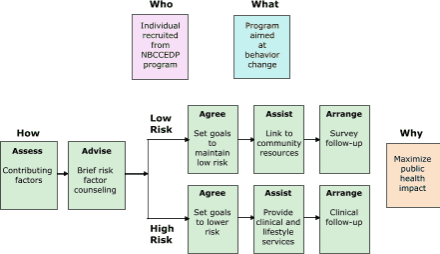
Example of an individual behavior change model, WISEWOMAN, United States.

For the practitioner, CDC's state and tribal partners, and CDC staff, knowing how to deliver a program is often the most difficult challenge. The various levels of the socioecologic model dictate different strategies for change. For example, the WISEWOMAN program endorses the 5A's strategy (Assess, Advise, Agree, Assist, Arrange) (25) for creating change at the individual level (Figure 2). Furthermore, allowing for multiple levels of stratification according to motivation and risk is consistent with the WISEWOMAN vision to create more appropriate and effective interventions.

**Table:** 

NBCCEDP indicates National Breast and Cervical Cancer Early Detection Program

**Figure 3.**
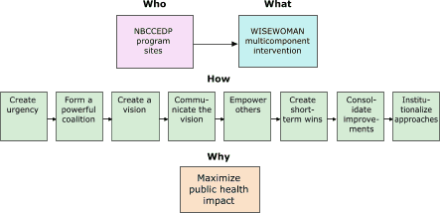
Example of an organizational, community, and policy change model, WISEWOMAN, United States.

At the organizational and community level, we will use the following steps advocated by Kotter (26) (Figure 3) to encourage adoption, faithful implementation, and maintenance of a project: 1) establish a sense of urgency, 2) form a powerful coalition, 3) create a vision, 4) communicate the vision, 5) empower others to act on the vision, 6) plan for and create short-term wins, 7) consolidate improvements and produce more change, and 8) institutionalize new approaches. To implement a WISEWOMAN project effectively, a local health organization (e.g., clinic, health department) will need, for example, to focus on preventive health care for under- and uninsured women as a key part of its mission, to use or adapt an intervention so that it is appropriate while remaining effective, to enhance use of community resources, and to create the organizational changes essential for implementing and sustaining the program over time.

**Table:** 

NBCCEDP indicates National Breast and Cervical Cancer Early Detection Program

Creating community and policy changes using Kotter's approach means first establishing a sense of urgency among community leaders about providing financially disadvantaged populations with access to resources and ensuring social and environmental support through such activities as creating walkable environments in the lowest-income neighborhoods. For policy changes, the initial task of creating a sense of urgency can involve issues such as universal access to preventive health services and the expansion of successful health promotion programs statewide.

## Conclusion

When it enters its implementation phase (Phase III), WISEWOMAN will be moving in some new directions. To extend reach and reduce costs, the program will help projects design interventions based on degrees of risk and levels of motivation and will ensure that projects have access to resources for promoting community health. These strategies are also likely to increase the effectiveness of the program. For example, one strategy has CDC providing evidence-based interventions and core elements through the Center of Excellence that conducts research on improving the public health impact of the WISEWOMAN program. Another strategy for increasing effectiveness is the provision of a clear framework and vision so that organizational, community, and other partners can make the case for why the program is important to their communities and what is needed to make it work.

Moving in a new direction always presents challenges. For WISEWOMAN, some of these challenges are ensuring that underfunded sites are not overburdened by having to conduct multiple interventions, measuring readiness to change when addressing multiple behaviors, packaging new interventions and updating old versions, monitoring projects for faithful implementation of core elements, and ensuring that women with normal screening values receive appropriate counseling and resources. Acknowledging these challenges, CDC looks forward to Phase III and hopes to be able to demonstrate the impact of these new directions through increased services to a greater number of women and a reduction in heart disease and stroke.

## Figures and Tables

**Table 1:** Challenges and Strengths Identified by States and Tribes Funded for Research and Nonresearch Projects, WISEWOMAN, Phases I and II, United States, 1995–2007

**Funded States**	**Challenges**	**Strengths**
**Phase I (1995-1998): Research**		
Arizona Massachusetts North Carolina	Difficulty integrating clinical and lifestyle interventions. Difficulty implementing research in public health settings. Need to extend reach beyond the individual. Difficulty developing organizational structure to focus on prevention of risk factors. Often biological outcomes improved in control groups, making determining the true effect of the lifestyle intervention difficult.	High rates of participation in lifestyle interventions. High number of women returning for 1-year rescreening. Comprehensive approach that addresses multiple health issues. Linkages to primary health care were strengthened. Innovative behavioral interventions provided. Lifestyle interventions demonstrated improvements in nutrition and physical activity.
**Phase II (1999-2007): Research and Nonresearch**		
**Research** Alaska (Southcentral Foundation) California Illinois Iowa North Carolina West Virginia **Nonresearch** Alaska (Southeast Alaska Regional Health Consortium) Connecticut Massachusetts Michigan Minnesota Missouri Nebraska South Dakota Vermont	Difficulty ensuring all women enroll in and complete lifestyle interventions. Difficultly ensuring rescreening at 10-14 months. Challenges in reaching the targeted number of annual screenings (2500). Benchmarks for performance indicators may have been unrealistic. Diabetes prevention and weight-loss strategies need strengthening. Lifestyle interventions adapted without evidence base.	Nonresearch projects provided greater flexibility and decreased research demands. More successful implementation of socioecologic model. High-performing sites provided opportunities for case studies to determine best practices. Many risk factors reduced significantly. Wide range of innovative interventions implemented. Some programs were able to go statewide.

**Table 2:** Clinical Guidelines that Support the Four WISEWOMAN Components, United States

**Component**	Clinical Guidelines
Cardiovascular risk factor screening	Third Report of the Expert Panel on Detection, Evaluation, and Treatment of High Blood Cholesterol in Adults, 2001 (ATP-III) Seventh Report of the Joint National Committee on Prevention, Detection, Evaluation, and Treatment of High Blood Pressure, 2004 (JNC 7) Guide to Clinical Preventive Services, 2005
Lifestyle intervention	ATP-III, JNC 7, and Guide to Clinical Preventive Services, 2005 (for dietary counseling of adults with risk factors) Guide to Community Preventive Services, 2001 (for Tobacco Quit Lines) Guide to Community Preventive Services, 2001 (for physical activity programs adapted for individual needs)
Treatment and medication	ATP-III, JNC 7
Rescreening for monitoring and evaluation	Guide to Clinical Preventive Services, 2005

ATP indicates Adult Treatment Panel.
